# Detecting transcriptionally active regions using genomic tiling arrays

**DOI:** 10.1186/gb-2006-7-7-r59

**Published:** 2006-07-19

**Authors:** Gabor Halasz, Marinus F van Batenburg, Joelle Perusse, Sujun Hua, Xiang-Jun Lu, Kevin P White, Harmen J Bussemaker

**Affiliations:** 1 Department of Biological Sciences, Columbia University, 1212 Amsterdam Avenue, New York, NY, 10027 USA; 2 Integrated Program in Cellular, Molecular and Biophysical Studies, Columbia University, 630 w. 168^th ^Street, New York, NY, 10032 USA; 3 Bioinformatics Laboratory, Academic Medical Center, University of Amsterdam, Meibergdreef 15, 1105 AZ Amsterdam, The Netherlands; 4 Department of Genetics, Yale University School of Medicine, 333 Cedar Street, PO Box 208005, New Haven, CT, 06520-8005, USA; 5 Department of Ecology and Evolutionary Biology, Yale University, 165 Prospect Street, PO Box 208106, New Haven, CT, 06250-8106, USA; 6 Center for Computational Biology and Bioinformatics, Columbia University, 1130 St. Nicholas Avenue, New York, NY, USA

## Abstract

We have developed a method for interpreting genomic tiling array data, implemented as the program TranscriptionDetector. Probed loci expressed above background are identified by combining replicates in a way that makes minimal assumptions about the data. We performed medium-resolution *Anopheles gambiae *tiling array experiments and found extensive transcription of both coding and non-coding regions. Our method also showed improved detection of transcriptional units when applied to high-density tiling array data for ten human chromosomes.

## Abstract 2

A new method for designing and integrating genomic tiling array data is described and applied to Anopheles and human arrays.

## Background

A complete understanding of an organism's biology requires identification of the complete set of RNA transcripts it expresses. Elucidating this 'transcriptome' has proven challenging for two reasons. First, even when a complete genome sequence is available, it has proven difficult to define the exact location and number of protein-coding genes [1]. Second, many transcripts are non-coding RNAs, which are thought to play a largely regulatory role, and are often active at relatively low levels, or in a tissue-specific manner. Expressed sequence tag (EST) sequencing and similar techniques will, therefore, often fail to detect them.

To fully catalog transcripts, several groups have used genomic microarrays, which assay expression with probes spaced more or less evenly along the genome [2-15]. These tools have higher sensitivity than EST sequencing, and provide a high-throughput way of measuring RNAs from different samples and cellular contexts. Whole-genome array studies of *Arabidopsis thaliana *[12,14], *Drosophila melanogaster *[13], *Saccharomyces cerevisiae *[4,10], *Oryza sativa *[8], *Mus musculus *[5] and *Homo sapiens *[2,3,6,7,9,11,15] all detect a great deal of transcription outside known protein-coding regions.

Despite the usefulness and recent popularity of whole-genome arrays, to date there is no standard way to perform such experiments or analyze their data [16]. Existing studies vary, among others, in their method of finding a threshold above which transcripts are considered to be expressed, in their choice of negative controls (if any) to obtain this threshold, and in their manner of combining information from multiple arrays. One feature that is usually shared, however, is the inference of transcriptional activity based on the signal intensities of multiple adjacent probes [2-9,11,15,17].

Various approaches are also used to account for background intensity (cross-hybridization to probes by partially complementary transcripts), probe sequence features that systematically bias signal measurements, and variability in the range of intensities between different arrays. Several studies have explicitly modeled signal intensities to distinguish signal from background noise. These models incorporate parameters for transcript concentration and probe-specific affinities [18,19] and array- and dye-associated variability in signal intensity [20], or explain signal intensity for a probe as a function of its sequence using statistical and thermodynamic models [21-25]. They usually differentiate between signal arising from hybridization of cognate transcript to the probe (specific hybridization) and signal arising from cross-hybridization. Finally, normalization procedures have been developed to remove non-biological variability between replicate microarray experiments [26].

In this paper, we introduce a strategy for designing and interpreting genome-wide tiling experiments, the final result of our analysis being a list of probed loci that are putatively expressed. Like some other methods [3,6,7,10,14], we make use of negative control probes that represent non-specific background hybridization to evaluate the significance of expression of individual probed loci. However, we combine information from replicates in a way that makes minimal assumptions about the distribution of signal intensities and avoids putting a threshold on individual replicates. In addition, we model the dependence of non-specific hybridization on probe sequence; subtracting the systematic bias explained by these models greatly improves our ability to detect transcripts. For high-density arrays, the signal of neighboring probes can be combined to take advantage of the fact that the same transcript will contribute to the intensity of multiple probes, but this is not essential to our approach, which can, therefore, be successfully applied to low-density tiling array data as well.

## Results

### Correcting for the effect of probe sequence on non-specific hybridization

**Figure 1:**
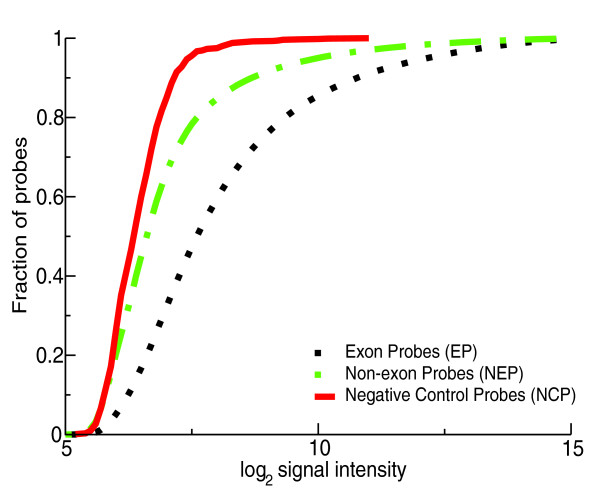
Signal intensity distributions of probes measuring annotated *A. gambiae *exons (EPs), non-exon regions (NEPs) and negative controls (NCPs). Cumulative distribution functions of signal intensities for these probe populations are shown for a representative channel.

Each of our arrays contained 76,782 probes interrogating annotated exons of *Anopheles gambiae *(exon probes (EPs)), 94,469 non-exon probes (NEPs), and 1,000 negative control probes (NCPs). As expected, the signal intensity distribution of EPs is shifted to the right of the NCP distribution (Figure 1). NEPs exhibit a similar albeit less pronounced shift, indicating that a substantial fraction of the non-coding regions are expressed above background. However, these differences may be partly explained by differences in probe sequence composition between the populations. Several studies have addressed the effect of probe sequence on signal intensity and developed tools to infer underlying transcript abundances using this information [21-25]. We also use a sequence-based model that reduces this non-biological variability in signal. However, since our goal is to infer which probed loci are transcribed at all (and not, for example, to determine which of two transcripts is more abundant), a relatively simple model dealing exclusively with background suffices for our purposes. If the null hypothesis that the signal intensity for a given probe can be fully explained by cross-hybridization and random noise is rejected, we conclude that this is due to hybridization of cognate transcript.

**Figure 2:**
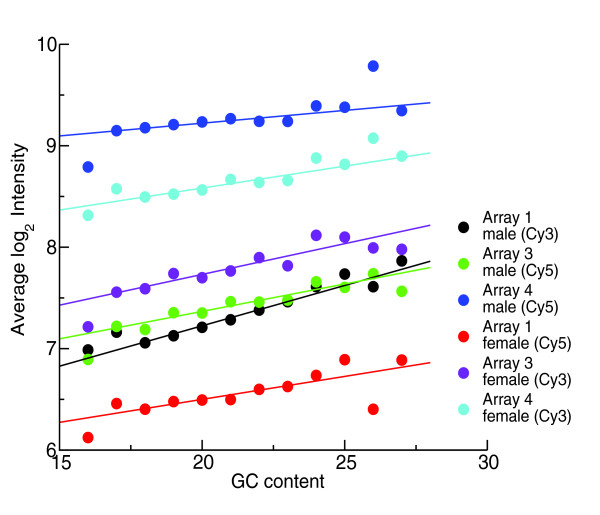
Linear dependence of signal intensity on GC content for negative control probes. Average log_2 _signal intensity of NCPs with indicated GC content, for six channels.

Since NCPs were designed as concatenations of 12-mers not found anywhere in the *A. gambiae *genome (see Materials and methods), their signal intensities can be considered as background only. This enables us to search for a relationship between probe sequence and background intensity. One such feature that needs to be accounted for if the signal intensities are to faithfully reflect transcript abundance is GC content. High GC content is associated with strong hydrogen bonding and an increased propensity to 'catch' cross-hybridizing RNA transcripts, which tend to be GC rich as well. This leads to a positive correlation between the signal intensity of a probe and its GC content (Figure 2), as had been previously observed for Affymetrix arrays [25,27].

To determine the best way to correct for probe sequence bias, we tested a number of different sequence models, ranging from a simple GC content model to a fully position-specific sequence model, which is an adaptation of [23,25,27]. Negative control probe intensities were fit independently for each 'channel' (that is, each unique combination of array and dye). The fraction of the NCP intensity variance that can be explained in terms of probe sequence ranges from 3% for the GC content model to 17% for the position-specific model (Table 1). We observed considerable variation in the model parameters between channels (data not shown), presumably due to channel-specific differences in labeling or synthesis. Each model fit was used to normalize the intensity of all probes to that of a reference probe in which all four bases are equally likely at any position (see Materials and methods). Correcting probe intensities by accounting for sequence bias did not substantially change the distribution of the three probe populations (supplementary Figure 1 in Additional data file 6). However, as discussed below and shown in the last column of Table 1, even the relatively modest reduction in variance of the NCP probe intensities achieved by the model-based probe sequence correction has a profound effect on the number of probed regions found to be transcribed. We decided to use the 'Full Position-specific' model for all our subsequent analysis.

### Dealing with variation in signal intensity across channels

**Figure 3:**
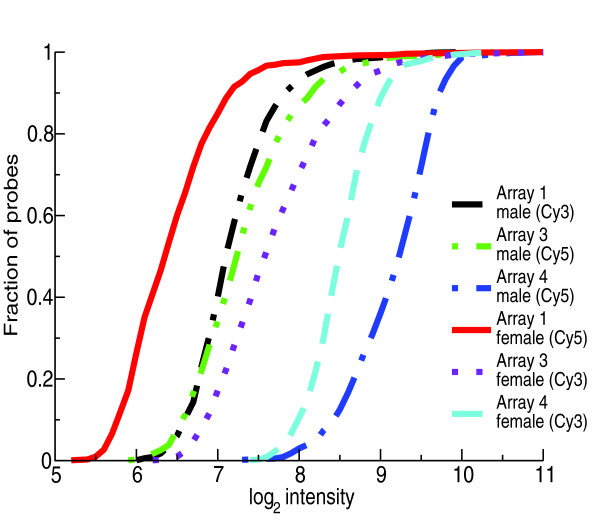
Variation between channels in the distribution of signal intensities for NCPs. Cumulative distribution functions of signal intensities for NCPs for different channels are shown.

Each probe has 10 signal intensity measurements associated with it (five labelings of each sex). Clearly, all of these values must be used in our determination of significance, but it is not obvious how to combine the 10 values in a parametric way. There is considerable variability in the distribution of intensities between microarrays, even when duplicate measurements (RNA samples from the same sex, labeled with the same dye) are considered (Figure 3; supplementary Figure 2 in Additional data file 6). In addition, there is considerable variation between different dye labelings on the same microarray, regardless of whether or not a probe sequence based signal correction has been applied to the data (supplementary Figure 3 in Additional data file 6). Because of these pronounced channel-specific effects, averaging of intensities across different experiments is not well justified.

Our approach solves this problem by pooling data from different channels in a fully non-parametric way, thereby avoiding any assumptions about how the different channels relate to each other. The only assumption we make is that of a monotonic relationship between signal intensity and transcript abundance for a given channel once the intensities have been corrected for probe sequence bias, as described above. The first step in this process assigns a channel-specific 'single-channel' *p *value to each probe, defined as the fraction of NCPs with signal intensity larger than that of the probe within the same channel. The second step combines the single-channel *p *values for each probe into a single 'multi-channel *p *value' (MCPV), reflecting the likelihood that the set of intensities observed for that probe can be interpreted as background signal. This approach obviates the need to explicitly model dye- and array-specific effects [20].

### Residual bias of negative control probes after sequence correction

In a classic approach to combining the result from multiple, independent statistical tests performed for the same feature, the product of individual *p *values is interpreted as a new test statistic, and transformed to a variable that is uniformly distributed between zero and one under the null assumption of independent tests for that feature, using a property of the *χ*^2 ^distribution [28] or an equivalent geometric approach [29]. We will refer to the resulting *p *value as a 'Fisher *p *value'.

**Figure 4:**
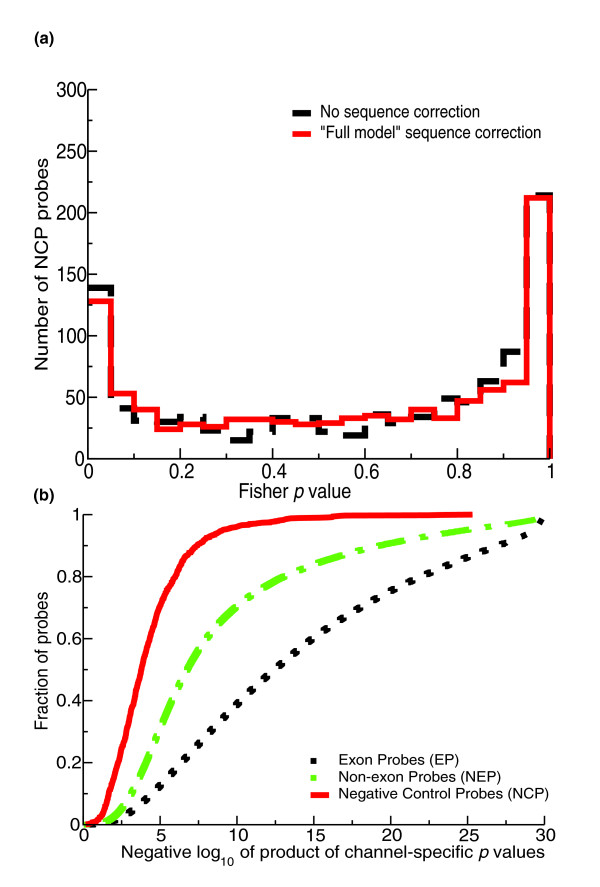
Combining information from replicate experiments. **(a) **Distribution of Fisher *p *values for NCPs, obtained by taking the product of channel-specific *p *values and comparing it to the product of random numbers drawn from a uniform distribution (see [28]). The solid line corresponds to probe signal intensities that were first corrected for probe sequence bias using the Full Position-specific model (see Materials and methods); the dashed line corresponds to uncorrected intensities. **(b) **Distribution of the negative log of products of single-channel *p *values for different probe populations.

The single-channel *p *values for NCPs are by construction uniformly distributed. However, it is not clear that the model-based correction for probe sequence bias is capable of completely removing any probe-specific bias in NCP intensity across channels. As Figure 4a shows, the Fisher *p *values obtained by integrating the single-channel *p *values for each NCP across channels are far from uniformly distributed. The peak near zero (one) corresponds to negative control probes that consistently have a bias towards higher (lower) signal intensity. This probe-specific bias in signal intensity remains even after sequence correction. A plausible explanation of this residual bias is that each probe will receive cross-hybridization contributions to its background signal intensity from a highly specific subset of transcripts that is unique to each probe. The probe-specific intensity correlations across channels created in this way lead directly to the distribution observed in Figure 4a. Indeed, if we artificially create such correlations by simulating NCP signal intensities as a probe-specific random normal variate to which a probe and channel specific random variate with the same standard deviation is added, and then calculate Fisher *p *values, we obtain a curve that strikingly resembles Figure 4a (data not shown). The shape of Figure 4a also remains unchanged when we repeat our analysis after removing the top and bottom 10% of NCPs as ranked by Fisher *p *value, indicating that the bias is not limited to a small subset of outlier probes. Explicit modeling of cross-hybridization between a probe and all possible transcripts is possible [30], but beyond the scope of this paper. It is interesting that while our sequence model only takes into account the probe sequence and is, therefore, not able to parameterize this probe-specific contribution to the background signal, the Fisher *p* values nevertheless reveal the existence of a probe-specific bias in the residual NCP intensities.

### Multi-channel *p *values: integrating evidence for transcription across channels

The existence of a subtle correlation between channels, presumably due to specific off-target hybridization, makes it impossible to use Fisher *p *values to integrate single-channel *p *values across multiple channels. However, we do want to integrate weak evidence for transcription from individual channels for the EP and NEP probes. This goal can be achieved by first computing the product of single-channel *p *values (derived from the NCP intensity distribution) for both NCP and EP/NEP probes. Multi-channel *p *values (MCPV) for EP/NEP are then defined as the fraction of NCPs with a *p *value product smaller than that for the probe in question (see Materials and methods). Comparison of Figure 1 with Figure 4b shows the increased separation between NCP, NEP, and EP distributions when evidence for transcription is integrated across channels.

### Application to low-density genomic array data for mosquito

**Figure 5:**
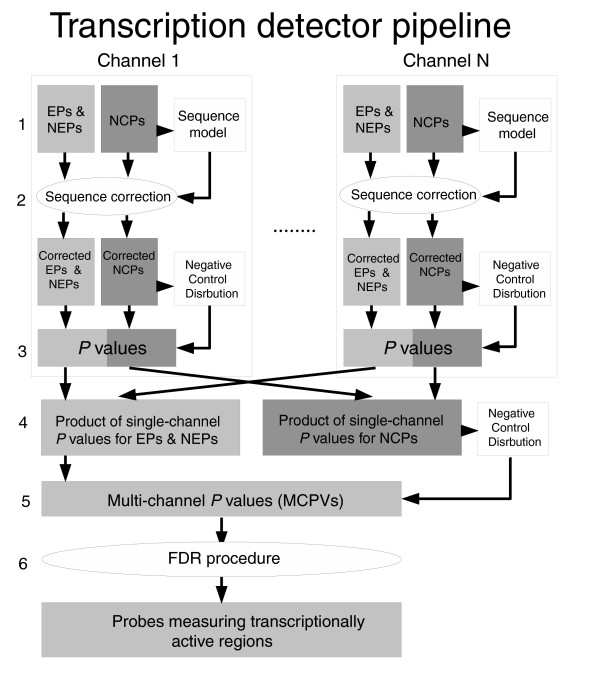
Schematic overview of the TranscriptionDetector data processing pipeline. First, a model accounting for the effect of probe sequence on non-specific binding is fit to the (log-transformed) NCP signal intensities ('step 1') and used to correct the intensity for all probes ('step 2'); a separate model is fit for each channel. For each probe, we then derive a *p *value reflecting the likelihood that its signal intensity belongs to the background distribution represented by the NCPs ('step 3'); these *p *values are calculated separately for each channel, and each channel-specific *p *value is treated as the outcome of an independent experiment. A multi-channel statistic equal to the product of *p *values across all channels is computed for each probe ('step 4'). In analogy with step 3, the distribution of this statistic for the NCPs only is then used to assign a MCPV to the other probes ('step 5'). To control for multiple hypothesis testing, a FDR procedure is used, and each probed locus is designated as transcribed or not transcribed ('step 6').

The MCPVs defined above are by construction uniformly distributed between zero and one for NCPs. They can, therefore, be considered to be *bona fide p *values that can be used as the basis for a false discovery rate procedure to obtain a list of putatively transcribed probed loci. To this end, we created a computer program called TranscriptionDetector that implements the pipeline detailed in Figure 5. It is available for download [31]. Given probe sequences and signal intensities for a set of identically designed arrays, TranscriptionDetector returns a list of probed loci expressed above background. Running it on the *A. gambiae *data set described above, we found that 26% of NEP and 51% of EP probes detect transcriptionally active loci.

### Application to high-density human tiling array data

On high-resolution tiling arrays, where probes are spaced closely together, a given transcript will contribute to the signal intensity of multiple consecutive probes. The more probes with a low MCPV we encounter in a given genomic region, the more confident we are that the region is transcribed. This reasoning is in direct analogy with that used to derive MCPVs in the first place: instead of integrating evidence across channels, we now wish to integrate evidence across adjacent probes. We achieved this by adding a 'smoothing' step, in which the MCPV of each probe is replaced by the Fisher *p *value obtained by combining its MCPV with that of its nearby neighbors. It is crucial that only non-overlapping neighboring probes be included in this neighborhood set, to guarantee the statistical independence of the various MCPVs that are being combined.

We compared the results of our method to that obtained by Cheng *et al*. [3] in their analysis of 10 human chromosomes using 25 base-pair (bp) probes at 5 bp resolution. This study lacked NCPs specifically designed not to match any genomic region, so we used a set of 2,634 non-spiked-in bacterial probe pairs instead. When smoothing using *n *probes on either side of the central probe (that is, combining 2*n *+ 1 MCPVs), we found that performance increased up to *n *= 5 and then stabilized, so we settled on that value, which corresponds to a region of approximately 275 bp. Applying a threshold to the resulting smoothed MCPVs classifies each probe as 'expressed' or 'not expressed'. Optionally, we applied the 'minrun' and 'maxgap' criteria used by Cheng *et al*. [3] (see Materials and methods).

**Figure 6:**
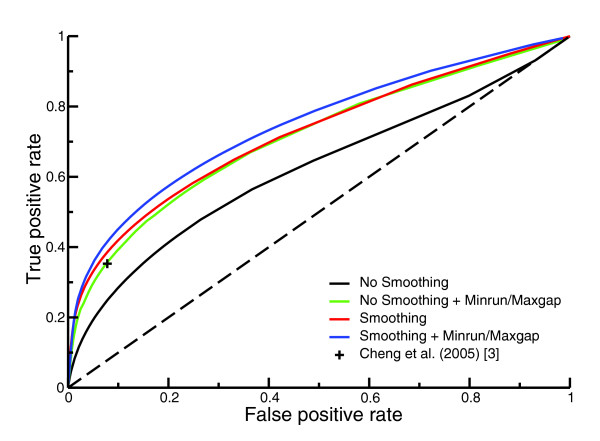
ROC curves showing true positive rate versus false positive rate relative to transcripts annotated in the UCSC database. The '+' symbol corresponds to the transfrags as defined by Cheng *et al*. [3]. Lines correspond to our algorithm as applied with/without neighborhood smoothing and with/without minrun/maxgap post-processing.

Figure 6 shows receiver operating characteristic (ROC) curves quantifying the sensitivity and specificity of our method at varying threshold value, using the genomic coordinates of 'known genes', mRNAs, and ESTs from the UCSC genome annotation database as a 'gold standard' (see Materials and methods). The point marked by the '+' symbol corresponds to the 'transfrags' reported by Cheng *et al*. [3], who applied a parametric smoothing procedure to their signal intensities, classified probes whose intensity exceeded a significance threshold in at least one of the replicates as 'expressed', and joined these positive probes into 'transfrags' using the minrun/maxgap procedure. The effectiveness of our non-parametric evidence integration across replicates is demonstrated by the fact that simply applying the minrun/maxgap criterion of Cheng *et al*. [3] after setting a MCPV threshold without the benefit of neighborhood smoothing already gives a similar performance (Figure 6, green line). When neighborhood smoothing (*n *= 5) is applied to the MCPVs (Figure 6, blue line) our method outperforms that of Cheng *et al*. [3], and the difference becomes even more pronounced when minrun/maxgap post-processing is applied: at the same false positive rate, the sensitivity for detecting the combined UCSC annotations is improved by 17%; at the same false negative rate, the specificity is improved by 37%. It is interesting to note that most of the improvement comes from the detection of ESTs (supplementary Figure 4 in Additional data file 6), which tend to be expressed at a lower level.

## Discussion

We have described a method for designing and interpreting genomic tiling array data that makes minimal assumptions about intensity distribution and variation between replicates. Combining the results from any number of hybridizations to a microarray whose design includes a set of NCPs, our algorithm assigns one MCPV to each probe, which can be used to determine which probed loci are transcriptionally active. Applying a signal intensity threshold only after the evidence from multiple channels has been combined enhances the sensitivity of our method. Including NCPs in the design of our microarray allowed us to quantitatively model the dependence of background signal intensity on probe sequence, without the need to simultaneously parameterize specific and non-specific contributions to signal intensity [21-24]. Reducing the variance of the NCP probe intensities by accounting for sequence bias using this model greatly increased the number of transcripts detected. More sophisticated sequence models could further improve our method's sensitivity.

The probe sequence correction (and for high-density tiling arrays the size of the smoothing neighborhood) is the only parametric component of our method. Beyond that, our algorithm uses a completely non-parametric approach to the problem of signal variability across channels; no assumptions are made about the distribution of signal intensities in each channel. Of course, there is the risk of decreased statistical power when using non-parametric methods when a parametric one would be justified. To address this issue explicitly, we calculated channel-specific Z-scores for each probe based on the mean and standard deviation of NCP intensity for each channel, and averaged these across channels for each probe. Alternatively, we performed quantile normalization [26], and then averaged intensities across channels for each probe. In both cases, the normalized and averaged intensities were subsequently used to derive a multi-channel *p *value for each probe. These parametric variants of our method gave results very similar to the approach defined in Figure 4. The Z-score-based approach identifies 96% to 99% of the probes reported in Table 1, while reporting 1% to 10% novel probes, depending on the sequence correction used; the corresponding ranges for the normalization-based scheme are 94% to 97% and 1% to 2%, respectively. In summary, this comparison shows that we are not sacrificing statistical power for the sake of simplicity.

Our initial attempt at integrating evidence across channels using Fisher *p *values uncovered a systematic probe-specific bias in NCP signal that persists across channels even after sequence correction (compare Figure 4a). It is interesting to note that this bias also manifests itself in the Z-score representation: if we compute the mean Z-score for each NCP probe across channels, the standard deviation of these means (0.638) is about twice as large as the inverse square root of 10, that is, the value that would be expected for 10 independent channels. Presumably, this effect is due to the sequence-specific partial hybridization between each control probe and a subset of the RNA transcripts present in the cell. This underscores the fact that, despite being designed to have at least three mismatches, NCPs are subject to substantial cross-hybridization. While it cannot be excluded that tiling probes experience a somewhat different spectrum of cross-hybridization contributions due to internal similarities within the genome, it seems reasonable to use the NCP intensities to estimate their variance.

The fraction of significantly expressed probed loci found for *A. gambiae *is considerably lower than the figure we reported for *D. melanogaster *in [13]. We attribute this discrepancy to an improvement in our analysis, specifically: a change in the definition of negative control probes; and our more stringent way of computing MCPVs. Repeating our analysis of *A. gambiae *using Fisher *p *values caused 43% of probed non-exonic loci and 75% of exonic loci to be classified as transcriptionally active, numbers that are very similar to those reported in [13].

Given the relatively sparse placement of probes on the *A. gambiae *arrays, and to avoid making assumptions about the structure or size of transcribed regions, we determined the significance of each probed locus independently of its neighbors. As we demonstrate using a high-density human data set, our method can be readily extended to take advantage of the fact that, at higher probe densities, a single transcript can contribute to the signal intensity of multiple adjacent probes. It is, therefore, useful for interpreting both high-density tiling arrays, where spatial dependencies can be exploited, and low-density arrays, where adjacent probes are too far apart to yield such information.

## Materials and methods

### Array design

The NASA Oligonucleotide Probe Selection Algorithm (NOPSA) was used to select optimal 36-mer probes measuring expression from EPs and NEPs. Coding and non-coding regions were identified based on annotations from the Ensembl database (file anopheles_gambiae_core_15_2). As a control for non-specific EP and NEP hybridization, 4,000 dodecanucleotides absent from the *A. gambiae *genome were identified computationally. NCPs were then formed by random concatenation of three such 12-mers, guaranteeing that each NCP had at least three mismatches relative to any 36 nucleotide stretch of the *Anopheles *genome. Five microarrays, each containing an identical set of 76,782 EPs, 94,469 NEPs and 1,000 NCPs were synthesized using Maskless array synthesizer (MAS) technology [32].

### Samples and hybridization

Three to five day old *A. gambiae *adults (G3 strain) were sorted by sex and homogenized in Trizol. Total RNA was isolated using Heavy phase lock gel columns (Invitrogen, Carlsbad, CA, USA) and polyadenylated RNA was extracted using oligodT chromatography columns (BioRad, Hercules, CA, USA). We labeled 3 μg of each experimental sample by chemical coupling of Cy3 or Cy5 dyes (Amersham, Piscataway, NJ, USA) to the aminoallyl nucleotide introduced during cDNA synthesis (Powerscript reverse transcriptase, BD Biosciences, Franklin Lakes, NJ, USA). Labeled samples were purified using RNeasy columns (Qiagen, Valencia, CA, USA) and hybridized overnight at 52°C to high density oligonucleotide microarrays. The arrays were scanned using an Axon scanner (Molecular Devices Corporation, Sunnyvale, CA, USA). Males were labeled twice with Cy3 and three times with Cy5; the reverse was done for females. Each array measured RNA from both sexes.

### Probe sequence bias correction

Five different models were used to relate NCP sequence to signal intensity (Table 1). The most basic is the 'GC model', which assumes a linear relationship between signal log-intensity and GC content. The 'Nucleotide-specific model' is slightly more complex, explaining the signal in terms of the representation of each base, not just G and C. The remaining two models take position dependencies into account by allowing different segments of the probe to make independent contributions to binding, and are described below.

The 'Bilinear model' derives both base- and position-specific parameters, under the assumption that these two variable types are independent. The signal intensity of each probe is then given by:

log⁡(I)=∑i=1nγi*βb(i)
 MathType@MTEF@5@5@+=feaafiart1ev1aaatCvAUfeBSjuyZL2yd9gzLbvyNv2Caerbhv2BYDwAHbqedmvETj2BSbqee0evGueE0jxyaibaiKI8=vI8tuQ8FMI8Gi=hEeeu0xXdbba9frFj0=OqFfea0dXdd9vqai=hGuQ8kuc9pgc9s8qqaq=dirpe0xb9q8qiLsFr0=vr0=vr0dc8meaabaqaciGacaGaaeqabaqadeqadaaakeaaciGGSbGaai4BaiaacEgacaGGOaGaamysaiaacMcacqGH9aqpdaaeWbqaaiabeo7aNnaaBaaaleaacaWGPbaabeaaaeaacaWGPbGaeyypa0JaaGymaaqaaiaad6gaa0GaeyyeIuoakiaacQcacqaHYoGydaWgaaWcbaGaamOyaiaacIcacaWGPbGaaiykaaqabaaaaa@4777@

where *γ*_*i *_is the weight for position *i *along the probe, *β*_*b *_is the weight for base *b*, *b(i) *is the base at position *i*, and *n *is the length of the probe. The values for the two sets of model parameters were determined by iterating between regression of *γ *and *β *until convergence.

The 'Full Position-specific model' combines the base and position weights into a single parameter *δ*_*i*,*b*, _reflecting the weight associated with having base *b *at position *i*. The signal log-intensity is then simply given by:

∑i=1nδi,b(i)
 MathType@MTEF@5@5@+=feaafiart1ev1aaatCvAUfeBSjuyZL2yd9gzLbvyNv2Caerbhv2BYDwAHbqedmvETj2BSbqee0evGueE0jxyaibaiKI8=vI8tuQ8FMI8Gi=hEeeu0xXdbba9frFj0=OqFfea0dXdd9vqai=hGuQ8kuc9pgc9s8qqaq=dirpe0xb9q8qiLsFr0=vr0=vr0dc8meaabaqaciGacaGaaeqabaqadeqadaaakeaadaaeWbqaaiabes7aKnaaBaaaleaacaWGPbGaaiilaiaadkgacaGGOaGaamyAaiaacMcaaeqaaaqaaiaadMgacqGH9aqpcaaIXaaabaGaamOBaaqdcqGHris5aaaa@3FA3@

This last model is essentially that of [23], who explained most of the variance in signal intensity with weights associated with a particular base at a particular position, and found that terms modeling features of secondary structure were less important. Other studies have used very similar models, but parameterize the positional dependence for each base as a polynomial [27] or using a spline [25].

### Computing Fisher *P *values for putatively independent channels

For each probe *k*, we first computed a test statistic *τ*_*k *_equal to the product of all single-channel *p *values *P*_*kc *_:

τk=∏c=1nPkc
 MathType@MTEF@5@5@+=feaafiart1ev1aaatCvAUfeBSjuyZL2yd9gzLbvyNv2Caerbhv2BYDwAHbqedmvETj2BSbqee0evGueE0jxyaibaiKI8=vI8tuQ8FMI8Gi=hEeeu0xXdbba9frFj0=OqFfea0dXdd9vqai=hGuQ8kuc9pgc9s8qqaq=dirpe0xb9q8qiLsFr0=vr0=vr0dc8meaabaqaciGacaGaaeqabaqadeqadaaakeaacqaHepaDdaWgaaWcbaGaam4AaaqabaGccqGH9aqpdaqeWbqaaiaadcfadaWgaaWcbaGaam4AaiaadogaaeqaaaqaaiaadogacqGH9aqpcaaIXaaabaGaamOBaaqdcqGHpis1aaaa@3FB9@

where *c *labels the channel and *n *is the total number of channels. Fisher *p *values were then computed as the probability that uniformly distributed independent random variables would yield a product of *p *values as high as that observed for a given probe. This probability is given by:

Fn(τ)=τ∑i=0n−1(−ln⁡τ)ii!
 MathType@MTEF@5@5@+=feaafiart1ev1aaatCvAUfeBSjuyZL2yd9gzLbvyNv2Caerbhv2BYDwAHbqedmvETj2BSbqee0evGueE0jxyaibaiKI8=vI8tuQ8FMI8Gi=hEeeu0xXdbba9frFj0=OqFfea0dXdd9vqai=hGuQ8kuc9pgc9s8qqaq=dirpe0xb9q8qiLsFr0=vr0=vr0dc8meaabaqaciGacaGaaeqabaqadeqadaaakeaacaWGgbWaaSbaaSqaaiaad6gaaeqaaOGaaiikaiabes8a0jaacMcacqGH9aqpcqaHepaDdaaeWbqaamaalaaabaGaaiikaiabgkHiTiGacYgacaGGUbGaeqiXdqNaaiykamaaCaaaleqabaGaamyAaaaaaOqaaiaadMgacaGGHaaaaaWcbaGaamyAaiabg2da9iaaicdaaeaacaWGUbGaeyOeI0IaaGymaaqdcqGHris5aaaa@4B4C@

See [29] for details.

### Multi-channel *p *values and false discovery rate procedure

Since cross-hybridizing transcripts invalidate the independence assumption, MCPVs were ultimately used in our procedure. These were obtained by comparing the *τ *statistic (as defined above) for each probe to a null distribution composed of the *τ*-values for the NCPs. A significance threshold was derived using a false discovery rate (FDR) procedure [33], using an FDR of 5%. Briefly, MCPVs were ranked in strictly increasing order: *P*_1 _≤ *P*_2_...≤ *P*_*n*_. The largest *i *for which:

Pi≤inα
 MathType@MTEF@5@5@+=feaafiart1ev1aaatCvAUfeBSjuyZL2yd9gzLbvyNv2Caerbhv2BYDwAHbqedmvETj2BSbqee0evGueE0jxyaibaiKI8=vI8tuQ8FMI8Gi=hEeeu0xXdbba9frFj0=OqFfea0dXdd9vqai=hGuQ8kuc9pgc9s8qqaq=dirpe0xb9q8qiLsFr0=vr0=vr0dc8meaabaqaciGacaGaaeqabaqadeqadaaakeaacaWGqbWaaSbaaSqaaiaadMgaaeqaaOGaeyizIm6aaSaaaeaacaWGPbaabaGaamOBaaaacqaHXoqyaaa@3A6B@

where *α *= 0.05, represents the largest MCPV that is still significant. Probes with MCPV less than or equal to P_*i *_are, therefore, considered to detect loci expressed above background.

### Evidence integration for adjacent probes on high-density tiling arrays

For each probe, Fisher *p *values were calculated over its MCPVs and those of up to *n *upstream and *n* downstream probes. If there were fewer than 2*n *probes within 30 × (*n*) nucleotides of the central probe, only these were used in the calculation. Because overlapping probes are not independent, only completely non-overlapping probes were used. The Fisher *p *value itself was calculated in exactly the same way as for putatively independent channels - the test statistic is now:

τk=∏i=k−nk+nPi
 MathType@MTEF@5@5@+=feaafiart1ev1aaatCvAUfeBSjuyZL2yd9gzLbvyNv2Caerbhv2BYDwAHbqedmvETj2BSbqee0evGueE0jxyaibaiKI8=vI8tuQ8FMI8Gi=hEeeu0xXdbba9frFj0=OqFfea0dXdd9vqai=hGuQ8kuc9pgc9s8qqaq=dirpe0xb9q8qiLsFr0=vr0=vr0dc8meaabaqaciGacaGaaeqabaqadeqadaaakeaacqaHepaDdaWgaaWcbaGaam4AaaqabaGccqGH9aqpdaqeWbqaaiaadcfadaWgaaWcbaGaamyAaaqabaaabaGaamyAaiabg2da9iaadUgacqGHsislcaWGUbaabaGaam4AaiabgUcaRiaad6gaa0Gaey4dIunaaaa@42BC@

where *k *labels the central probe being evaluated and *P*_*i *_is the MCPV for probe *i*.

### Analyisis of Affymetrix high-density human tiling array data

Affymetrix CEL expression files, CDF probe annotatation files, and negative control probe data were downloaded from [34]. An array-specific *p *value was computed for each tiling path probe by comparing its log(PM/MM) value to a negative control distribution of non-spiked-in bacterial probe pairs. *P *values for different replicates were combined into a single MCPV, which in turn were smoothed as described in the previous section, using *n *= 5. To keep our comparison with Cheng *et al*. [3] focused, we did not sequence correct probe intensities and applied the same minrun (50 bp) and maxgap (30 bp) criteria as described in that study (probes above a certain smoothed MCPV threshold were considered positive; if two such positive probes were within maxgap bases of each other, all probes between them were also considered positive; a contiguous stretch of positive probes must be at least minrun bases in length, otherwise the probes in the 'failed' run are considered negative).

### ROC curve analysis

Transcribed regions ('transfrags') predicted by Cheng *et al*. [3] (cytosolic/polyA+ samples only) were downloaded from [34], and a union was taken across all cell lines. UCSC genome annotation files for ESTs, mRNAs, and annotated ('known') genes were downloaded from [35]. Probes overlapping any part of these UCSC regions were taken to be our gold standard, relative to which sensitivity and specificity were calculated. For Cheng *et al*. [3], the predicted probes were considered to be those overlapping their predicted transfrags. For our analysis, predicted probes were obtained as described in the previous section, using a range of MCPV thresholds.

### Data deposition

Raw expression data for the present study has been submitted to the NCBI Gene Expression Omnibus as series GSE5196.

## Additional data files

The following additional data are available with the online version of this paper. Additional data file 1 contains probe sequence and raw signal intensities for exon probes. Additional data file 2 contains probe sequence and raw signal intensities for non-exon probes. Additional data file 3 contains probe sequence and raw signal intensities for negative control probes. Additional data file 4 contains genomic coordinates for regions measured by exon probes. Additional data file 5 contains genomic coordinates for regions measured by non-exon probes. Additional data file 6 contains four supplementary figures: supplementary Figure 1 demonstrates that signal variability between different probe populations on the same channel is not explained by probe sequence composition; supplementary Figure 2 shows Q-Q plots for NCP signal intensities in different channels, showing that these have heterogeneous and non-normal distributions; supplementary Figure 3 demonstrates that signal variability between negative control probes on different channels is not explained by probe sequence composition; supplementary Figure 4 has two ROC curves showing true positive rate versus false positive rate relative to **(a) **mRNA and **(b) **EST transcripts annotated in the UCSC database (the '+' symbol corresponds to the transfrags as defined by Cheng *et al*. [3]; and lines correspond to our algorithm as applied with/without neighborhood smoothing and with/without minrun/maxgap post-processing).

## Supplementary Material

Additional date file 1 Probe sequence and raw signal intensities for exon probes Click here for file

Additional date file 2 Probe sequence and raw signal intensities for non-exon probes Click here for file

Additional date file 3 Probe sequence and raw signal intensities for negative control probes Click here for file

Additional date file 4 Genomic coordinates for regions measured by exon probes Click here for file

Additional date file 5 Genomic coordinates for regions measured by non-exon probes Click here for file

Additional date file 6 Supplementary Figure 1 demonstrates that signal variability between different probe populations on the same channel is not explained by probe sequence composition; supplementary Figure 2 shows Q-Q plots for NCP signal intensities in different channels, showing that these have heterogeneous and non-normal distributions; supplementary Figure 3 demonstrates that signal variability between negative control probes on different channels is not explained by probe sequence composition; supplementary Figure 4 has two ROC curves showing true positive rate versus false positive rate relative to **(a) **mRNA and **(b) **EST transcripts annotated in the UCSC database (the '+' symbol corresponds to the transfrags as defined by Cheng *et al*. [3]; and lines correspond to our algorithm as applied with/without neighborhood smoothing and with/without minrun/maxgap post-processing) Click here for file

## Figures and Tables

**Table 1:** Summary of sequence correction models

Model	Formalism	Number of parameters	Average R^2^	Average adjusted R^2^	Number of transcriptionally active regions
Uncorrected	NA	NA	NA	NA	47,463
GC	log *I *= *β*_0 _+ *β*_*GC*_(*N*_*C *_+ *N*_*G*_)	2	0.0293	0.0284	52,384
Nucleotide-specific	log *I *= *β*_0 _+ *β*_*A*_*N*_*A *_+ *β*_*C*_*N*_*C *_+ *β*_*G*_*N*_*G*_	4	0.0412	0.0373	53,982
Bilinear	log *I *= *β*_0 _+ ∑i=136δiβb(i) MathType@MTEF@5@5@+=feaafiart1ev1aaatCvAUfeBSjuyZL2yd9gzLbvyNv2Caerbhv2BYDwAHbqedmvETj2BSbqee0evGueE0jxyaibaiKI8=vI8tuQ8FMI8Gi=hEeeu0xXdbba9frFj0=OqFfea0dXdd9vqai=hGuQ8kuc9pgc9s8qqaq=dirpe0xb9q8qiLsFr0=vr0=vr0dc8meaabaqaciGacaGaaeqabaqadeqadaaakeaadaaeWaqaaGGaciab=r7aKnaaBaaaleaacaWGPbaabeaaaeaacaWGPbGaeyypa0JaaGymaaqaaiaaiodacaaI2aaaniabggHiLdGccqWFYoGydaWgaaWcbaGaamOyaiaacIcacaWGPbGaaiykaaqabaaaaa@4116@	41 = 36 + 4 + 1	0.0980	0.0604	61,731
Full Position-specific	log *I *= *β*_0 _+ ∑i=136δi,b(i) MathType@MTEF@5@5@+=feaafiart1ev1aaatCvAUfeBSjuyZL2yd9gzLbvyNv2Caerbhv2BYDwAHbqedmvETj2BSbqee0evGueE0jxyaibaiKI8=vI8tuQ8FMI8Gi=hEeeu0xXdbba9frFj0=OqFfea0dXdd9vqai=hGuQ8kuc9pgc9s8qqaq=dirpe0xb9q8qiLsFr0=vr0=vr0dc8meaabaqaciGacaGaaeqabaqadeqadaaakeaadaaeWaqaaGGaciab=r7aKnaaBaaaleaacaWGPbGaaiilaiaadkgacaGGOaGaamyAaiaacMcaaeqaaaqaaiaadMgacqGH9aqpcaaIXaaabaGaaG4maiaaiAdaa0GaeyyeIuoaaaa@3FF4@	109 = 36 × 3 + 1	0.1709	0.0703	71,400

Overview of the models used to relate probe sequence to signal intensity. The Full Position-specific model has the highest R^2 ^and also the highest adjusted R^2^, indicating that overfitting is not a concern. The rightmost column shows the number of probed loci classified as transcriptionally active, which varies greatly with the sequence model used. NA, not applicable.
