# Supplementary material for: Detecting transcriptionally active regions using genomic tiling arrays
Source: Genome Biol. 2006 Jul 19;7(7):R59. doi: 10.1186/gb-2006-7-7-r59 (PMC1779562; doi:10.1186/gb-2006-7-7-r59)
Supplement: Additional date file 6 — Supplementary Figure 1 demonstrates that signal variability between different probe populations on the same channel is not explained by probe sequence composition; supplementary Figure 2 shows Q-Q plots for NCP signal intensities in different channels, showing that these have heterogeneous and non-normal distributions; supplementary Figure 3 demonstrates that signal variability between negative control probes on different channels is not explained by probe sequence composition; supplementary Figure 4 has two ROC curves showing true positive rate versus false positive rate relative to (a) mRNA and (b) EST transcripts annotated in the UCSC database (the '+' symbol corresponds to the transfrags as defined by Cheng et al. [3]; and lines correspond to our algorithm as applied with/without neighborhood smoothing and with/without minrun/maxgap post-processing) [file gb-2006-7-7-r59-S6.pdf]

Figure S1

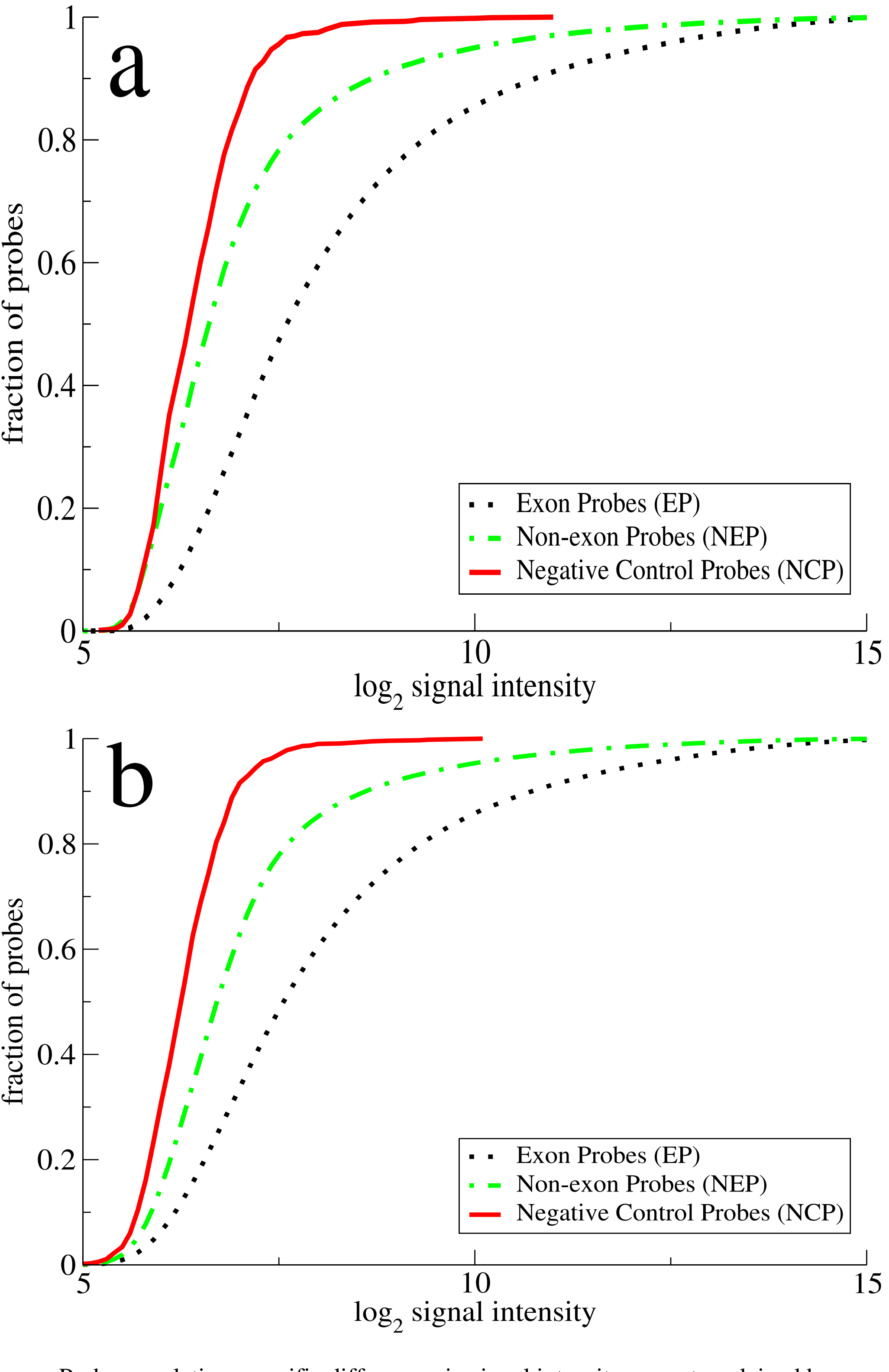

Probe population−specific differences in signal intensity are not explained by variation in probe sequence composition. Shown are cumulative distribution functions of signal intensities for annotated exons (EPs), non−exon regions (NEPs) and negative control probes (NCPs) in a representative channel either without (a) or with (b) correction using the Full Position−Specific Model. (Note that (a) is identical to Figure 1 in the main text.)

Figure S2

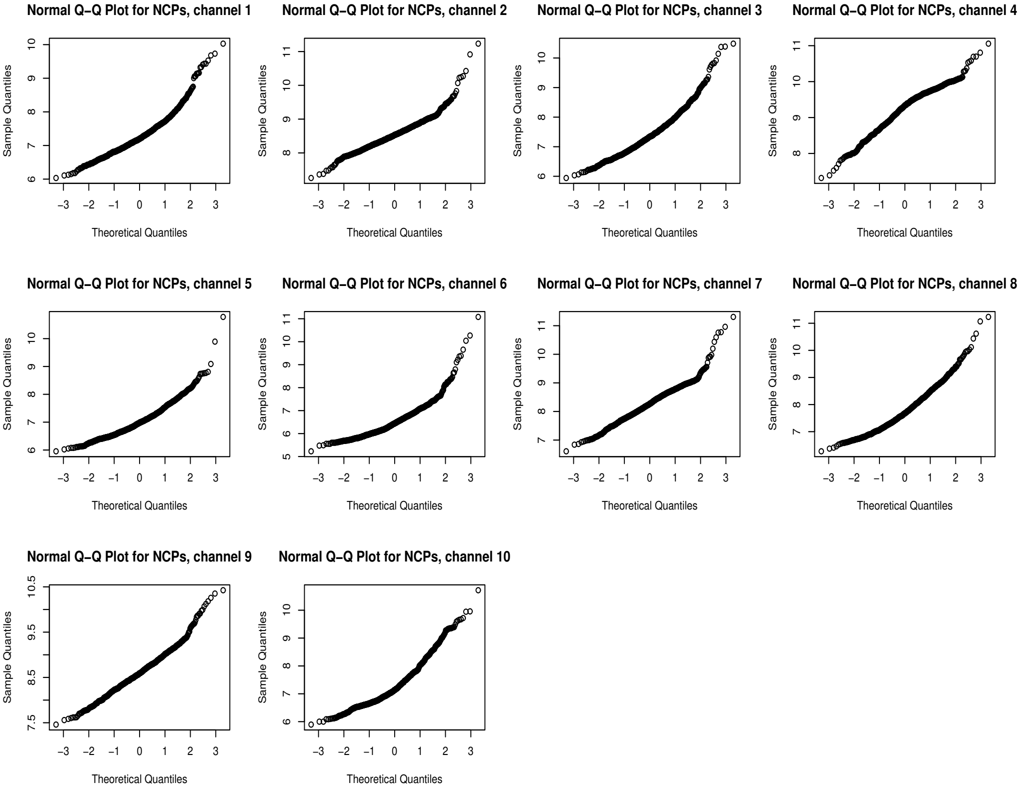

Quantile−Quantile plots comparing NCP signal intensities for different channels to a random normal distribution. All channels show deviations from normality.

Figure S3

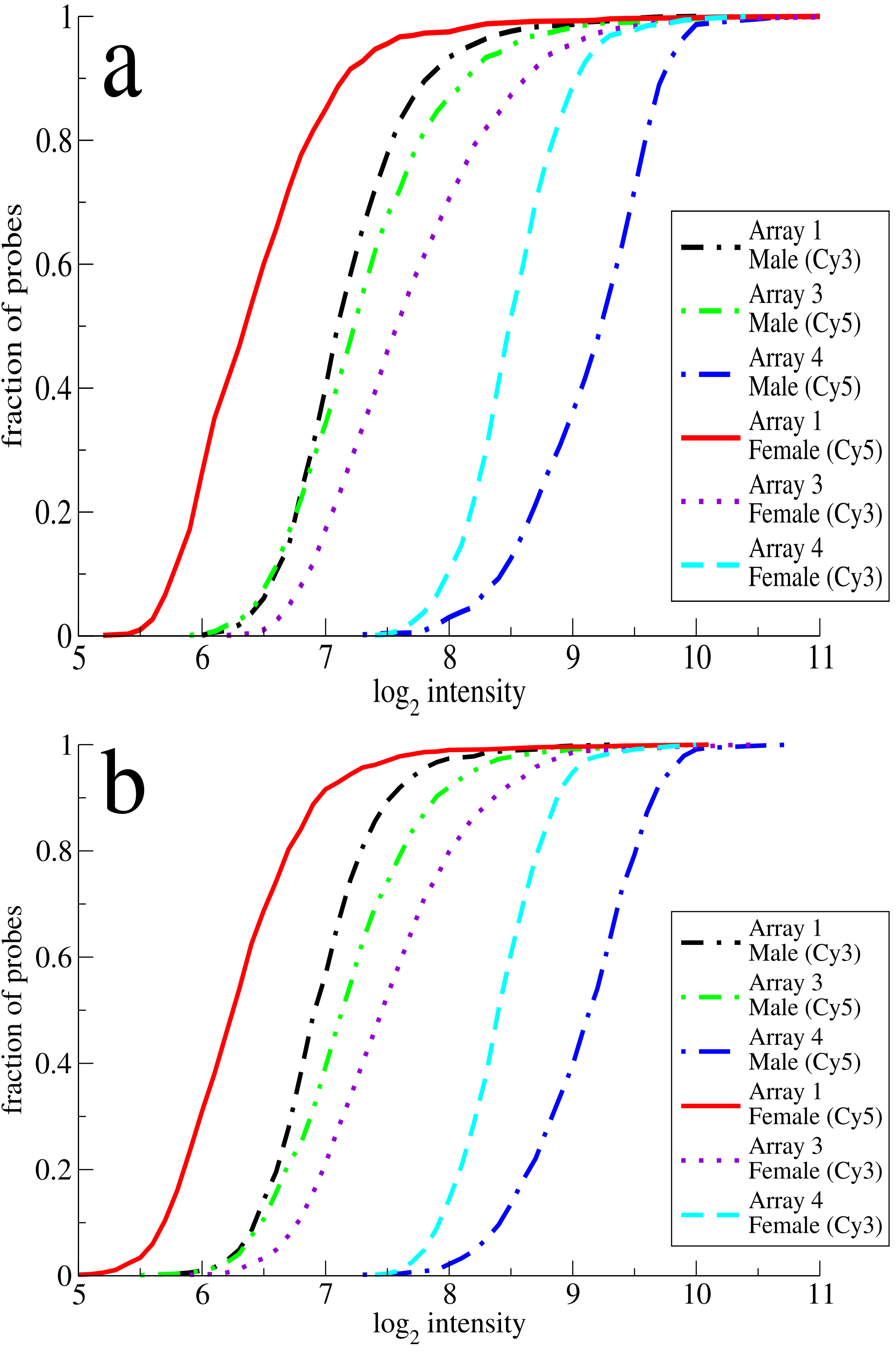

Variation between channels in the distribution of negative control probe (NCP) signal intensities remain after sequence correction. Cumulative distribution functions of signal intensities are shown for NCPs in different channels either without (a) or with (b) correction using the Full Position−Specific Model. (Note that (a) is identical to Figure 3 in the main text.)

Figure S4

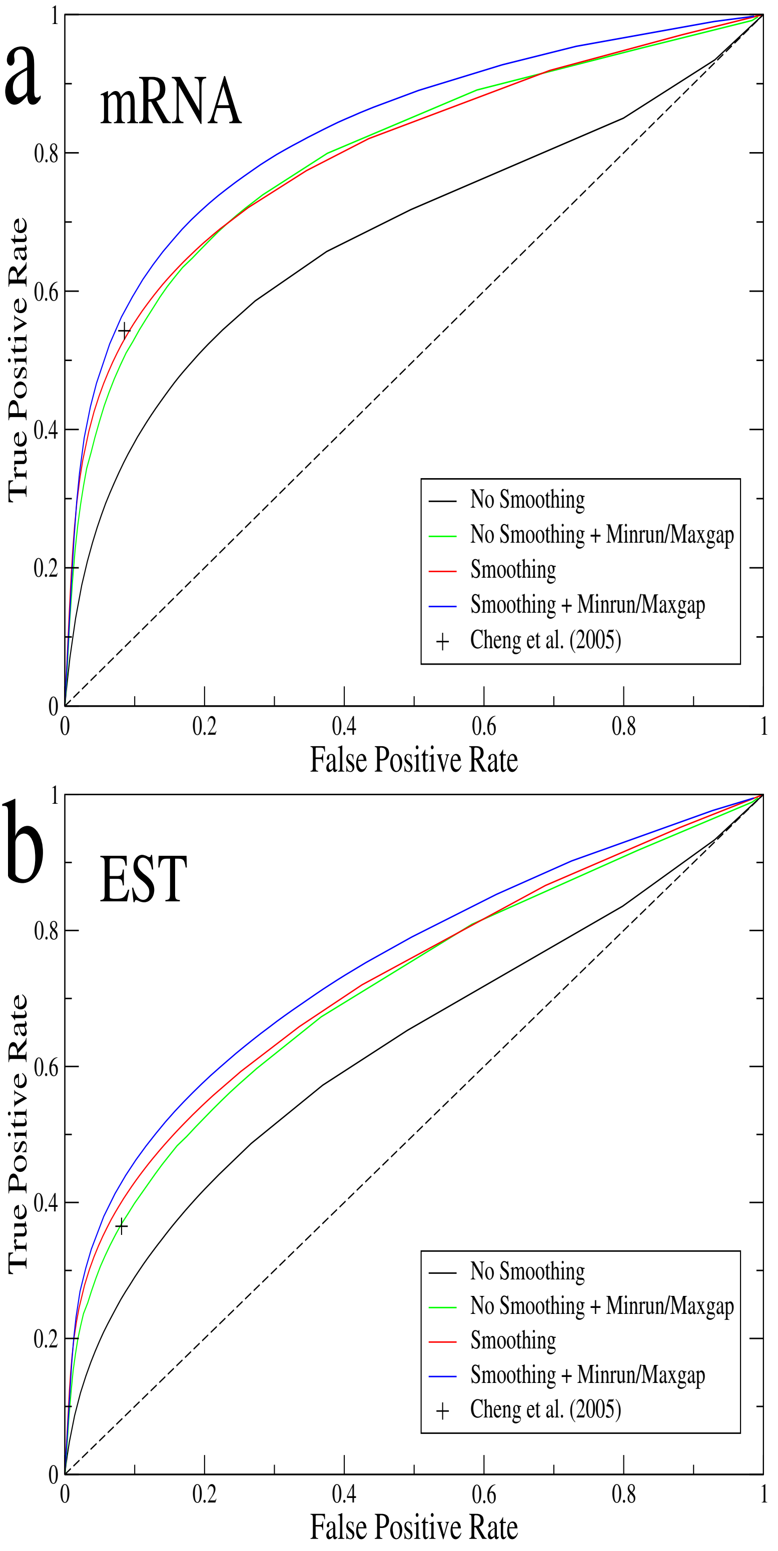

ROC curve showing true positive rate vs. false positive rate relative to mRNA (a) and EST (b) transcripts annotated in the UCSC database. The "+" symbol corresponds to the transfrags defined by Cheng et al. [10]. Lines correspond to our algorithm as applied with/without neighborhood smoothing and with/without minrun/maxgap post−processing.
